# Modulation of Immune Function in Rats Using Oligosaccharides Extracted from Palm Kernel Cake 

**DOI:** 10.1155/2017/2576921

**Published:** 2017-11-19

**Authors:** Mohammd Faseleh Jahromi, Parisa Shokryazdan, Zulkifli Idrus, Rohollah Ebrahimi, Fatemeh Bashokouh, Juan Boo Liang

**Affiliations:** ^1^Institute of Tropical Agriculture, Universiti Putra Malaysia (UPM), 43400 Serdang, Malaysia; ^2^Agriculture Biotechnology Research Institute of Iran (ABRII), East and North-East Branch, P.O. Box 91735 844, Mashhad, Iran; ^3^Faculty of Medicine, Universiti Teknologi MARA (UiTM), 40450 Shah Alam, Selangor Darul Ehsan, Malaysia

## Abstract

To investigate the prebiotic and immunomodulatory effects of PKC extract (OligoPKC) a total of 24 male rats were randomly assigned to three treatment groups receiving basal diet (control), basal diet containing 0.5% OligoPKC, or basal diet containing 1% OligoPKC for four weeks. We found that OligoPKC had no significant effect on the tested growth parameters. However, it increased the size of the total and beneficial bacterial populations while reducing pathogen populations. OligoPKC increased the concentration of immunoglobulins in the serum and cecal contents of rats. It also enhanced the antioxidant capacity of the liver while reducing lipid peroxidation in liver tissue. OligoPKC affected the expression of genes involved in immune system function in the intestine. Therefore, OligoPKC could be considered a potential mannan-based prebiotic for humans and animals due to its beneficial effects on the health and well-being of the model rats.

## 1. Introduction

Prebiotics were first identified by Gibson and Roberfroid [1] as “non-digestible food ingredients that stimulate the growth and/or activity of bacteria in the digestive system in ways benefiting health of the host.” Prebiotic is a general name for wide range of oligosaccharide (OS) compounds, including glucooligosaccharide, galactooligosaccharides, fructooligosaccharide, mannanoligosaccharide, xylooligosaccharide, and galactooligosaccharides, with different molecular structures. In the intestinal tract, prebiotics enhance populations of beneficial bacteria (such as lactic acid bacteria and bifidobacteria) [2]. In addition, prebiotics have many beneficial effects, such as reducing pathogen colonization in the intestine and cholesterol level of serum, enhancing immunoglobulin production, and improving metal absorption [3–5]. While these effects have been observed in various* in vitro* and* in vivo* studies, not every OS results in the same outcome. The prebiotic effects of OSs are dependent on different factors, including molecular structure of the OSs, profile of microorganisms in the intestine, and the health status of the individual. Thus, the beneficial effects of a specific OS cannot be extrapolated from those of other OSs, and it is important to investigate the prebiotic ability of every OS individually.

Diet can improve host resistance to infection by modulating immune function. Immunomodulation by prebiotics has been investigated in many studies [6–9]. Prebiotics and their fermentation products [primarily short chain fatty acids (SCFAs)] improve the function of gut-associated lymphoid tissue as well as the systemic immune system [6]. Previous studies have reported the various effects of OSs and mannanoligosaccharides (MOSs) on different animal models but most have been beneficial in terms of the growth and feed conversion ratio (FCR) of animals [10–13]. In the poultry industry, among the oligosaccharides frequently used, MOSs are the most common, and the two most popular commercial prebiotics (FOS and Bio-Mos®) have been reported to improve the health and performance of the birds [14].

Palm kernel cake (PKC) is the main by-product of the palm oil industry in the tropical countries Malaysia, Indonesia, Thailand, and Colombia. Malaysia is the world's second largest palm oil producer, with an annual production of more than 2.39 million tons of PKC [15]. Our earlier studies showed the beneficial effect of PKC (20% of diet) on enhancing the* Lactobacillus* and* Bifidobacterium *populations while reducing the* E. coli* and enterobacteria populations in the cecum of broilers [16]. The overall beneficial effect of PKC on intestinal microbiota was higher than that of Bio-Mos, a commercial prebiotic (Alltech Inc., Nicholasville, KY, USA). Based on this finding, we suggested that this effect was due to the OSs found in PKC. The extraction and characterization of the OS content of PKC extract (OligoPKC) have been performed by our laboratory (unpublished data). We found that mannose is the primary monosaccharide (MSC) found in OligoPKC, suggesting that its biological function in animal tissue might be the same as that of Bio-Mos [more than 733 published trials [17]] or other mannan oligosaccharide- (MOS-) based prebiotics, which contain mainly mannose in oligomer form. Therefore, the objective of the present study was to investigate the prebiotic and immunomodulatory effects of OligoPKC in a rat model.

## 2. Materials and Methods

Preparation of the OligoPKC was previously described [18]; carbohydrate determination using high-performance liquid chromatography (HPLC) was also described in detail in our previous study [19].

The animal experiment was approved by the Ethics Committee of the Universiti Putra Malaysia and was in compliance with the National Research Council's Guide for the Care and Use of Laboratory Animals [20]. A total of 24 male rats (six- to seven-week-old Sprague-Dawley) were used in this study. They were procured from the Faculty of Biotechnology and Bimolecular Science, Universiti Putra Malaysia, Selangor, Malaysia. The animals were individually housed in wire-topped plastic cages (47 cm length × 35 cm width × 20 cm height) of appropriate space and with free access to tap water and standard rodent diet (Specialty Feeds, Glen Forrest, WA, Australia). The animals were randomly assigned to three groups of eight rats each. After acclimatization for 7 days, each group of animals received one of the following diets: (i) basal diet (control); (ii) basal diet containing 0.5% OligoPKC (L); and (iii) basal diet containing 1% OligoPKC (H). For the preparation of treatment diets, OligoPKC was dissolved in water to concentrations of 0, 5, and 10% (w/v). Then, 100 mL of each solvent (containing 0, 5, and 10% OligoPKC) was sprayed on 1 kg of standard diet, and the water content of the diets was removed by drying at 60°C for 24 h. Physical parameters, such as body weight, feed intake, and mortality (if any), were recorded throughout the four-week experimental period. Blood and cecal samples were collected for serum immunoglobulin determination and cecal microbial quantification. In addition, jejunum and liver tissues were collected and stored in liquid nitrogen for gene expression studies. The antioxidant capacity and lipid peroxidation of the liver were also determined.

The levels of immunoglobulin A, E, G, and M (IgA, IgE, IgG, and IgM) in the serum and IgA in the cecal content were measured using commercial ELISA kits for rats (Cusabio Biotech Co., Ltd., Wuhan, China) according to the manufacturer's protocols.

The populations of selected microbiota (i.e., total bacteria,* Lactobacillus*,* Bifidobacterium, Enterococcus, *Enterobacteriaceae, and* E. coli*) in cecal samples were determined using the method described in our previous study [19] with specific primers listed in Table 1.

In the present study, the expression of several genes (Table 2) involved in the function of the immune system and in mannose metabolism was investigated. Gene expression analysis was conducted according to the method described previously [21].

2,2′-Azino-bis (3-ethylbenzothiazoline-6-sulfonic acid) (ABTS) and ferric reducing ability of plasma (FRAP) assays were used to determine the antioxidant capacity of the liver samples. The ABTS method followed that described by Tsai et al. [22] with some modifications explained in the previous study [18]. The FRAP assay was carried out according to the method developed by Benzie and Strain [23] with minor modifications described previously [18]. For both the ABTS and FRAP methods, different concentrations of Trolox (5 to 50 *μ*g/mL) were used to prepare a standard curve, and the results were expressed as *μ*g Trolox/g sample.

Lipid oxidation in the liver samples was measured using thiobarbituric acid-reactive substances (TBARS) according to the method developed by Lynch and Frei [24] and modified by Mercier et al. [25]. One gram of each liver sample was homogenized in 4 mL of 0.15 M KCl and 0.1 mM BHT. Each homogenized sample (200 *μ*L) was incubated with 1% (w/v) 2-thiobarbituric acid in 50 mM NaOH (0.25 mL) and 2.8% (w/v) trichloroacetic acid (0.25 mL) in a water bath at 95°C for 60 min until a pink colour developed. After cooling, 1 mL of distilled water and 3 mL of n-butyl alcohol were added, and the mixture was vortexed. The mixtures were then centrifuged at 5000 rpm for 10 min. The absorbance of the supernatant was read against an appropriate blank at 532 nm using a spectrophotometer (Secomam, Domont, France). The TBARS were calculated from the standard curve of 1,1,3,3-tetraethoxypropane and were expressed as *μ*g malondialdehyde (MDA) per g of liver tissue.

Experimental data were analysed by one-way ANOVA using SAS software [26] version 9.2. Tukey's Honest Significant Difference (HSD) post hoc test was performed to indicate which groups were significantly different from others. Differences were considered significant if *P* < 0.05.

## 3. Results

### 3.1. Monosaccharide and OS Contents of OligoPKC

The analysis of OligoPKC using HPLC showed that this product contains 62.74% (dry matter) OS with degrees of polymerization (DP, the number of monomeric units in an oligomer or polymer molecule) ranging from two to eight and 14.82% (dry matter) MSC. The rest of the components consist of mostly OSs of higher molecular weight, fatty acids, and proteins. Because previous research has shown that smaller OSs have higher prebiotic effects [27], we chose to focus on the prebiotic and immunomodulatory effects of the OSs in OligoPKC with lower molecular weights (DP2 to DP8).

### 3.2. Growth Parameters

Although the L and H levels of OligoPKC had no significant effect on growth parameters such as final body weight, daily and total weight gain, feed intake, and FCR, supplementation with this product numerically improved (i.e., reduced) the level of FCR (Table 3).

### 3.3. Immunoglobulin Concentrations

We observed a marked effect of OligoPKC on the level of immunoglobulin in the serum and cecum of rats (Table 4). Supplementation of OligoPKC at a high level (H group, 1%) significantly (*P* < 0.05) increased the concentrations of IgA (in the serum and cecal contents) and IgG (in the serum). However, a low level of OligoPKC supplementation (L group, 0.5%) only increased the concentration of IgA in the cecal content and IgG in the serum. There were no significant differences between two treatment groups compared to control in terms of IgE and IgM serum concentrations.

### 3.4. Microbial Quantification of Cecal Content

The quantified cecal populations of total bacteria in the cecal content, beneficial bacteria (*Lactobacillus*,* Bifidobacterium,* and* Enterococcus*), and pathogenic bacteria (*Enterobacteriaceae* and* E. coli*) are presented in Table 5. In both the L and H groups, OligoPKC increased the total bacterial populations of* Lactobacillus *and* Enterococcus* in comparison with the control group. However, the* Bifidobacterium* population was numerically higher in the L treatment group and was significantly (*P* < 0.5) higher in the H treatment group compared to the control. OligoPKC supplementation at both levels (0.5 and 1%) reduced the* Enterobacteriaceae* and* E. coli *populations. OligoPKC significantly reduced the pH of the cecal content of rats (*P* < 0.05).

### 3.5. Gene Expression

**Figure 1:**
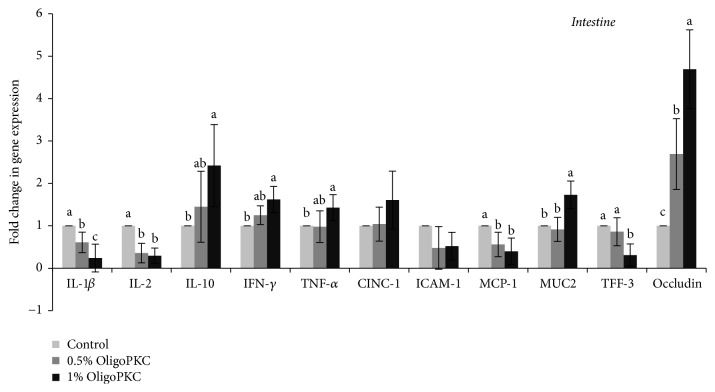
Effect of dietary supplementation with OligoPKC on the expression of genes involved in immune system function in the jejunum of rats. Bars represent the mean of eight replicates. Samples labelled with different letters differ significantly (*P* < 0.05). Error bars indicate the standard deviation. IL: interleukin; IFN: interferon; TNF: tumour necrosis factor; MCP: monocyte chemoattractant protein; CINC: cytokine-induced neutrophil chemoattractant; ICAM: intercellular adhesion molecule; MUC: mucin; TFF: trefoil factor.

Supplementation with OligoPKC in both the L and H treatment groups downregulated the expression of interleukin- (IL-) 1*β*, IL-2, and monocyte chemoattractant protein-1 (MCR-1) in the jejunum tissue in comparison with the control group (Figure 1). Trefoil factor- (TFF-) 3 was downregulated only in the H group. In contrast, the expression levels of IL-10, interferon-*γ* (IFN-*γ*), tumour necrosis factor (TNF-*α*), and mucin 2 (MUC2) in the H group and occludin in both the L and H groups were upregulated following treatment with OligoPKC in comparison with the control (Figure 1). OligoPKC did not affect the expression of intercellular adhesion molecule-1 (ICAM-1) and cytokine-induced neutrophil chemoattractant-1 (CINC-1) (*P* > 0.05).

**Figure 2:**
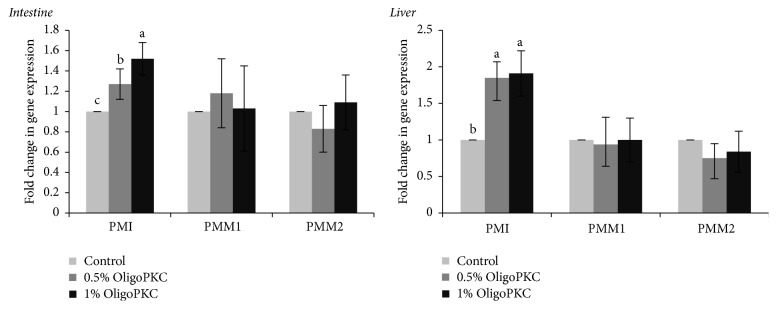
Effect of OligoPKC dietary supplementation on the expression of genes involved in mannose metabolism in the jejunum and liver of rats. Bars represent the mean of eight replicates. Samples labelled with different letters differ significantly (*P* < 0.05). Error bars represent the standard deviation. PMI: phosphomannose isomerase; PMM: phosphomannomutase.

The effects of OligoPKC on the expression of genes involved in mannose metabolism in the jejunum and liver of rats are presented in Figure 2. OligoPKC significantly increased the expression of phosphomannose isomerase (PMI) in the liver (in both the L and H groups) and intestine (only in the H group) (*P* < 0.05). It had no effect on the phosphomannomutase (PMM) genes in either the liver or intestine (*P* > 0.05) (Figure 2).

### 3.6. Antioxidant Activity

**Figure 3:**
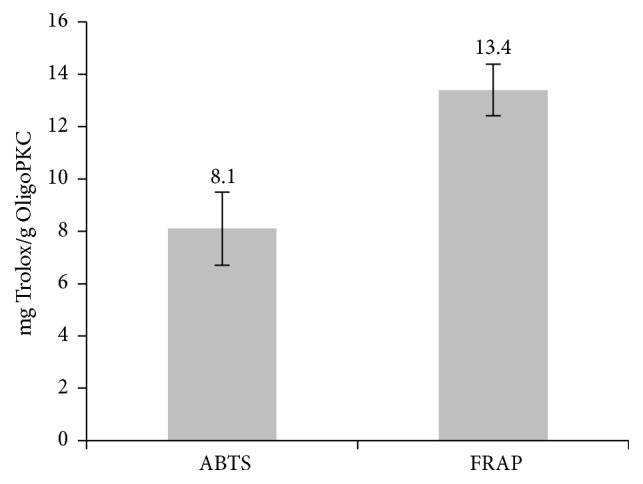
Antioxidant activity of OligoPKC, as determined using the ABTS and FRAP methods. Bars represent the mean of three replicates. Error bars indicate the standard deviation.

**Figure 4:**
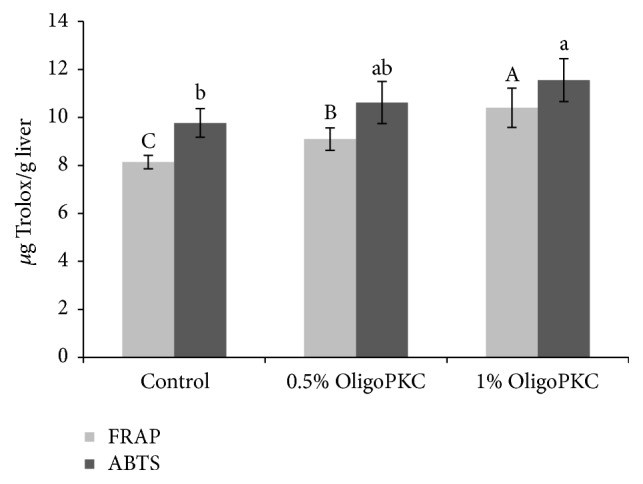
Effect of OligoPKC dietary supplementation on the antioxidant capacity of the liver in rats, as assessed using the ABTS and FRAP methods. Bars represent the mean of eight replicates. Samples labelled with different letters differ significantly (*P* < 0.05). Error bars indicate the standard deviation.

**Figure 5:**
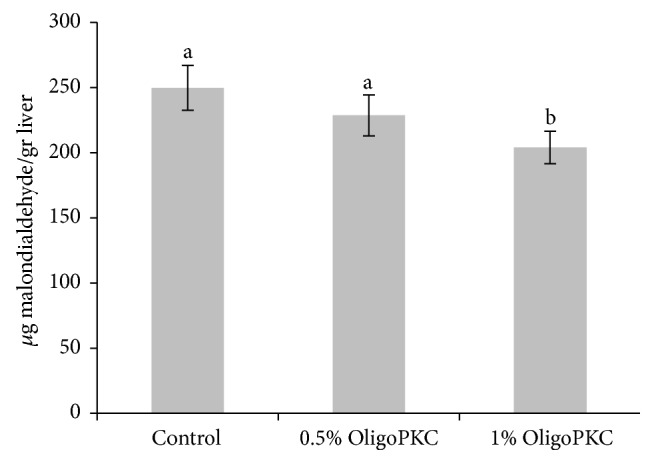
Effect of OligoPKC dietary supplementation on lipid peroxidation in the liver of rats according to the malondialdehyde method. Bars represent the mean of eight replicates. Samples labelled with different letters differ significantly (*P* < 0.05). Error bars indicate the standard deviation.

The antioxidant activity of OligoPKC determined using the ABTS and FRAP methods was 8.1 and 13.4 mg Trolox/g OligoPKC, respectively (Figure 3). High levels of OligoPKC enhanced the antioxidant capacity of the liver (Figure 4) and resulted in a reduction in lipid peroxidation in liver tissue (Figure 5).

## 4. Discussion

The results of our present and previous studies have shown that OligoPKC contains considerable amounts of OS with a DP of two to eight (59.39% of dry mater) [28]. Hence, we investigated the prebiotic and immunomodulation effects of OligoPKC using a rat model.

In the present study, dietary supplementation of OligoPKC had no significant effect on body weight, body weight gain, feed intake, and FCR of the animals. However the FCR of both the L and H groups were numerically lower than that of the control group. In terms of feed intake, although some studies have reported that prebiotics can modify the blood concentrations of gut-derived hormones such as glucagon-like peptide-1 and ghrelin, which might be involved in appetite regulation and reducing feed intake in rats [29–31], an effect on feed intake was not observed in the present study.

General health of the intestinal tract is enhanced by prebiotics supplementation via the improvement of the diversity and population size of its microbiota. It is thought that a healthy intestinal tract improves the efficiency of nutrient utilization by the host. Gut microbiota plays an important role in gut health and in turn in the general health and well-being of the host. The beneficial effects of gut microbes are linked to their fermentation profiles and the end-products of fermentation, as well as their capacity for producing vitamins and antioxidant compounds (reduction equivalents). They also defend against potentially pathogenic competitors and exchange molecular signals between different genera/species and epithelial intestinal cells [2]. The gastrointestinal tract (GIT) provides a complex ecosystem for its microbiota. Hence, to ensure a healthy gut for the host, it is necessary to manipulate the gut microbiota to increase the number of beneficial microorganisms while decreasing the population of harmful microbes. This is the expectation for all efficient prebiotics and represents one of the most important criteria in the evaluation of a compound as a prebiotic [1, 3]. Several studies conducted on animal models and humans as well as present study have shown that prebiotics shift the intestinal microflora toward beneficial populations by increasing the number of beneficial bacteria such as bifidobacteria and lactobacilli and by reducing the pathogens such as* E. coli*,* Salmonella,* and enterobacteria present in the gut. However, the results of different prebiotics are not very consistent [2, 11, 32, 33]. Increases in the population of beneficial bacteria following treatment with OligoPKC indicate that OSs derived from OligoPKC which are not digested in the small intestine were transferred to the large intestine and fermented by beneficial bacteria. Prebiotics are substrates for the metabolism and growth of beneficial microbes (lactic acid bacteria), and in turn these microorganisms could inhibit pathogen colonization through competitive exclusion and the production of antibacterial metabolites such as organic acid, hydrogen peroxide, and bacteriocins [34, 35]. The reduction of intestinal pH by OligoPKC due to the production of organic acids by lactic acid bacteria as well as the increase in the* Lactobacillus, Lactococcus, *and bifidobacteria (organic acid-producing species) populations in treatment groups provides support for this theory.

Researchers had examined the ability of mannose, a monomer MOS, to inhibit* Salmonella* infections. It was reported that* Salmonella* bound to mannose via type 1 fimbriae (finger-like projections), thereby reducing pathogen colonization of the intestinal tract [36]. The same mechanism may be underlying the reduction of* Enterobacteriaceae* and* E. coli* populations in the present study, so that OligoPKC could bind to the pathogens in the intestinal lumen, thereby blocking the adhesion of these bacteria to epithelial cells. However, additional research is required to verify this suggestion.

OligoPKC caused a significant increase in the concentrations of IgA and IgG and a numerical increase in IgE and IgM. This could be due to a direct effect of OligoPKC on the expression of genes responsible for cytokine production; at the same time, this could be explained by an indirect effect of OligoPKC on the immune system via the enhancement of populations of beneficial microbes such as* Lactobacillus*,* Lactococcus, *and bifidobacteria. It has been suggested that such beneficial bacteria and their metabolic products are able to affect the expression of cytokine genes to enhance immune function.

In the case of indirect effects of prebiotics on the immune system, the mere presence of a particular microbial genus or species, or a relative decrease of other microbes, triggered by supplementation with prebiotics could change the collective immunointeractive profile of the microbiota in the gut. For instance, Ito et al. [29] showed that the IgA concentration in the serum of rats following supplementation with fructans (inulin-type fructans) was positively correlated with the cecal* Lactobacillus *count. Szabó et al. [37] investigated the influence of the probiotic bacterium* Enterococcus faecium* on weanling pigs to determine its effect on* Salmonella typhimurium* infection. Interestingly, probiotic treatment resulted in increased production of specific antibodies (serum IgG, IgM, and IgA) against* S. typhimurium*. Another study showed that administering probiotics (*Bacillus subtilis *Bs964*, Candida utilis* BKM-Y74, and* Lactobacillus acidophilus* LH1F) can lead to increased IgA levels in the lumen; IgA-, IgM-, and IgG-producing cells; and T cells in cecal tonsils [38]. However, it is important to consider that probiotics alone (i.e., without prebiotics) may not reach their maximum effect. For example, feeding a combination of* Lactobacillus rhamnosus* GG,* Bifidobacterium lactis* Bb12, and prebiotics (i.e., inulin enriched with oligofructose) enhanced IgA secretion in the ileum. In contrast, probiotics alone had only a slight immunomodulatory effect [6].

We found that OligoPKC (1% of diet) upregulated the expression of cytokine genes IL-10, IFN-*γ*, and TNF-*α*, while it downregulated the expression of cytokine genes IL-1*β* and IL-2 in the intestine of rats. This could be the mechanism underlying the increase in immunoglobulin concentrations, both in the serum and in cecal contents of the animals. It has been reported that the direct effects of prebiotics could be the result of their effects on the expression of genes involved in immune system function [39]. For instance, mice fed with FOS and inulin exhibit an improved response to the* Salmonella* vaccine and at the first week after immunization exhibit increased production of cytokines such as IFN-*γ*, IL-12, and TNF-*α* in splenic cell cultures as well as an increase in* Salmonella*-specific blood IgG and faecal IgA. Hence, the FOS/inulin mixture stimulated host mucosal immunity to produce a greater response to the* Salmonella* vaccine [40]. An effect of prebiotics on the expression of genes involved in human immune system function has also been reported. Vulevic et al. (2008) investigated the effect of a mixture of galacto-OSs (galactans) on the immune systems of healthy elderly volunteers. They reported that the consumption of galactans (5.5 g/d) for 10 weeks significantly increased phagocytosis and production of the anti-inflammatory cytokine IL-10 while reducing production of the proinflammatory cytokines IL-1, IL-6, and TNF-*α* [41].

In the present study, expression of the IFN-*γ* gene in intestinal tissue was upregulated by supplementation of high (significant) and low (numerical) levels of OligoPKC, which could explain the increase in IgA in the serum and cecal contents of rats. IFN-*γ* is known to stimulate the expression of the secretory component of IgA (SIgA) by epithelial cells, which results in an increase in IgA production [40]. Nevertheless, Roller et al. [6] could not find any correlation between the expression of IFN-*γ* and the cecal SIgA concentration following prebiotic supplementation in rats, arguing against the potential role of this cytokine in stimulating SIgA [40].

Following secretion, the polymeric form of IgA is transported by its receptor (polymeric immunoglobulin receptor or pIgR) across the epithelium to the mucosal surface. Sollid et al. [42] reported that IFN-*γ* stimulates the expression of pIgR to mediate the transport of IgA by epithelial cells. Although we did not measure pIgR expression, upregulation of IFN-*γ* in the jejunum may in part explain why high cecal IgA concentrations were observed in rats fed OligoPKC.

Another possibility is that prebiotics could affect immunoglobulin concentrations indirectly by way of SIgA digestion in the cecum by bacterial species such as those of Clostridia, which have been shown to possess protease activity capable of degrading IgA. These proteases have an optimal pH within the neutral range [43]. Therefore, increases in the population of cecal lactobacilli and other acid-producing bacteria leading to reductions in pH may reduce the degradation rate of IgA and increase its secretion in the cecum [29].

Chemokines play a major role in selectively recruiting monocytes, neutrophils, and lymphocytes, as well as in inducing chemotaxis through the activation of G-protein-coupled receptors. The migration of monocytes from the blood stream across the vascular endothelium is required for routine immunological surveillance of tissues as well as for the response to inflammation. Chemokines such as MCP-1 and CINC-1 and adhesion molecule ICAM-1 are expressed in response to inflammation. In a study by Rodríguez-Cabezas et al. [44], inflamed colonic tissue in untreated colitic rats exhibited increased expression of MCP-1, CINC-1, and ICAM-1 in comparison with healthy rats.

Mucin 2 is the primary constituent of the colonic protective mucus layer, whereas TFF-3 is a bioactive peptide that is involved in epithelial protection and repair. The results of a study by Rodríguez-Cabezas et al. [44] revealed that FOS increased the expression of MUC2 and TFF-3 in rats receiving FOS in comparison with untreated control rats [44] in order to improve intestinal barrier function. The effect of prebiotics on upregulating the expression of TFF-3 and MUC2 was also reported by Daddaoua et al. [45]. Similarly, in the present study, high levels of OligoPKC significantly increased the expression of the MUC2 gene in the intestine, which could lead to improvement in the protective and functional effects of the mucosa. Such a result was not observed for TFF-3. Occludin is one of the major tight-junctional structural proteins that determine intestinal selective barrier function. The occludin level therefore reflects the destruction of gut tight-junctions by pathogens [46]. Cani et al. (2009) showed that prebiotic treatment (oligofructose) increased occludin mRNA in the jejunum segment of mice. Our findings also reveal the upregulation of occludin expression in the jejunum of rats following supplementation with OligoPKC.

Within cells, phosphomannose mutase (PMM) catalyses the conversion of mannose-6-phosphate to mannose-1-phosphate. High levels of PMM mRNA have been described in human liver, heart, brain, and pancreas [47, 48]. The biosynthesis of asparagine-linked glycans from glucose requires phosphomannose isomerase (PMI), which interconverts fructose-6-P and mannose-6-P. This reaction allows mannose and glucose to fuel either glycolysis or glycoconjugate synthesis. PMI mRNA is present in all examined human and mouse tissues and is more abundant in testis, brain, and heart [49]. Davis and Freeze [48] have found that mannose supplementation has little effect on the specific activity of PMM in different organs but that the specific activity of PMI in the brain, intestine, muscle, heart, and lung gradually increased more than twofold with increasing mannose intake. This is in agreement with our results, which showed that supplementation with OligoPKC upregulated the expression of PMI in both liver and intestine without any significant effect on PMM expression. Furthermore, changes in the expression of PMI in the liver support our hypothesis that the mannose content of OligoPKC can be transferred to the liver and can alter biological activity and gene expression in liver cells. In the present study, OligoPKC exhibited a high level of antioxidant activity, which could be due to its OS and small quantity of phenolic compound remained in the extract.

It has been reported that carbohydrates and carbohydrate-containing biomolecules can be considered true antioxidants, capable of scavenging reactive oxygen species (ROS) [50, 51]. According to Stoyanova et al. [50], ROS scavenging abilities of inulin and alternative natural sweeteners such as stevioside are superior scavengers of both hydroxyl and superoxide radicals, more effective than mannitol and sucrose. According to Morelli et al. [51], antioxidant activity of disaccharides (maltose and sucrose) is stronger compared to monosaccharides. According to an experiment conducted by Oskoueian et al. [52], the ethanolic extract from PKC contains high levels of fatty acids, phenolic compounds, sugar derivatives, and other organic compounds possessing high antioxidant activity. In their study, treating heat-induced hepatocytes with PKC extract (125 *μ*g/ml) resulted in a decrease in lipid peroxidation and an increase in antioxidant enzymatic activity in the cells. Our results are in agreement with this report of the high antioxidant activity of PKC extract; however, OligoPKC mainly consists of OSs and fewer phenolic compounds. Rats treated with supplemental OligoPKC exhibited a higher antioxidant capacity and lower lipid peroxidation in the liver compared to rats in the control group. Similarly, another study showed that mice treated with prebiotics exhibited decreased hepatic expression of inflammatory and oxidative stress markers in comparison with mice in the control group [53].

## 5. Conclusion

Investigation of the prebiotic and immunomodulatory effects of OligoPKC in a rat model revealed that although dietary supplementation had no significant effect on final body weight, daily and total weight gain, feed intake, and the feed conversion ratio of the rats, it improved the immune system function of the animals by controlling the expression of genes involved in immune system function, thereby enhancing the concentration of immunoglobulins in the serum and cecal contents. In turn, this increased the cecal population of beneficial bacteria and decreased the cecal population of pathogenic bacteria. OligoPKC supplementation also increased the antioxidant capacity of the liver and reduced lipid peroxidation in the liver. Due to these beneficial effects in the animal model, OligoPKC can be considered an efficient potential prebiotic for humans and animals.

## Figures and Tables

**Table 1:** Specific primers and annealing temperatures for target groups of bacteria.

Microorganism	Primer	Size of the amplified fragment (bp)	Annealing temperature (°C)
Total microbes	F-5′-CGGCAACGAGCGCAACCC-3′	145	55
	R-5′-CCATTGTAGCACGTGTGTAGCC-3′		
*Lactobacillus*	F-5′-CATCCAGTGCAAACCTAAGAG-3′	341	58
	R-5′-GATCCGCTTGCCTTCGCA-3′		
*Enterococcus *genus	F-5′-CCCTTATTGTTAGTTGCCATCATT-3′	144	50
	R-5′-ACTCGTTGTACTTCCCATTGT-3′		
*Bifidobacterium*	F-5′-GGGTGGTAATGCCGGATG-3′	440	60
	R-5′-TAAGCCATGGACTTTCACACC-3′		
*Escherichia coli*	F-5′-GTGTGATATCTACCCGCTTCGC-3′	82	50
	R-5′-AGAACGCTTTGTGGTTAATCAGGA-3′		
Enterobacteriaceae	F-5′-CATTGACGTTACCCGCAGAAGAAGC-3′	195	50
	R-5′-CTCTACGAGACTCAAGCTTGC-3′		
Total *Salmonella*	5′-TCGTCATTCCATTACCTACC-3′	119	50
	5′-AAACGTTGAAAAACTGAGGA-3′		
Clostridiaceae	F-5′-GAG TTT GAT CMT GGC TCA G-3′	552	55
	R-5′-CCC TTT ACA CCC AGT AA-3′		
*Campylobacter* spp.	F: 5-GGATGACACTTTTCGGAG-3	246	55
	R: 5-AATTCCATCTGCCTCTCC-3		

**Table 2:** Primer sequences and annealing temperatures used to examine gene expression.

Gene	Forward (5′→3′)	Reverse (5′→3′)	Annealing temperature (°C)
*β*-Actin	ACCCACACTGTGCCCATCTA	CGGAACCGCTCATTGCC	57
GAPDH	TGCACCACCAACTGCTTAGC	GGCATGGACTGTGGTCATGAG	60
PMI	AGTGTTCCCACTTTCCTGTG	CAGCTTTCCGTTAAAGGTGTC	57
PMM1	ATTGATCCTGAGGTATCAGCC	AATTGATCAAGTCCTGTAGGAGAND	57
PMM2	CTCTGTCTCTTTGACATGGA	CCCAGATGACCTTGAATATTCTG	57
IL-1*β*	CATCTTTGAAGAAGAGCCCG	AACTATGTCCCGACCATTGC	60
IL-2	TCCCCATGATGCTCACGTTTA	CATTTTCCAGGCACTGAAGATG	57
IL-10	CCTGCTCTTACTGGCTGGAG	GGCAACCCAAGTAACCCTTA	60
IFN-*γ*	GCCAAGTTCGAGGTGAACAAC	TAGATTCTGGTGACAGCTGGTGAA	57
TNF-*α*	GGATGAACACGCCAGTCGCC	CGAGTGACAAGCCCGTAGCC	60
MCP-1	CACTATGCAGGTCTCTGTCACG	CTGGTCACTTCTACAGAAGTGC	60
CINC-1	GGCAGGGATTCACTTCAAGA	GCCATCGGTGCAATCTATCT	60
ICAM-1	AGGTATCCATCCATCCCACA	AGTGTCTCATTGCCACGGAG	60
MUC2	GCTCAATCTCAGAAGGCGACAC	CCAGATAACAATGATGCCAGAGC	60
TFF3	ATGGAGACCAGAGCCTTCTG	ACAGCCTTGTGCTGACTGTA	60
Occludin	AAACCCGAAGAAAGATGGACC	TCACTTTGCCGTTGGAGGAG	60

GAPDH: glyceraldehyde phosphate dehydrogenase; PMI: phosphomannose isomerase; PMM: phosphomannomutase; IL: interleukin; IFN: interferon; TNF: tumour necrosis factor; MCP: monocyte chemoattractant protein; CINC: cytokine-induced neutrophil chemoattractant; ICAM: intercellular adhesion molecule; MUC: mucin; TFF: trefoil factor.

**Table 3:** Effect of OligoPKC on growth rate, feed intake, feed conversion ratio, and cecal pH in rats.

Parameter	Control	0.5% OligoPKC (L)	1% OligoPKC (H)
Initial body weight, g	109.18 ± 4.48	107.78 ± 6.01	107.79 ± 4.44
Final body weight, g	236.00 ± 4.33	236.27 ± 7.21	237.67 ± 9.52
Total gain in 4 weeks, g	126.82 ± 2.16	127.83 ± 4.91	129.88 ± 6.68
Daily gain, g	4.53 ± 0.08	4.57 ± 0.17	4.64 ± 0.24
Daily feed intake, g	12.56 ± 0.37	12.41 ± 0.30	12.42 ± 0.83
FCR, g/g	2.77 ± 0.097	2.72 ± 0.068	2.68 ± 0.095
Cecal pH	7.2 ± 0.4^a^	6.7 ± 0.2^b^	6.4 ± 0.3^b^

Values are the mean ± standard division of six replicates; FCR: feed conversion ratio. ^a-b^Means labelled with different superscripts within a row differ significantly (*P* < 0.05).

**Table 4:** Effect of OligoPKC on immunoglobulin level in serum and cecum.

Immunoglobulin	Control	0.5% OligoPKC (L)	1% OligoPKC (H)
IgA (*μ*g/g cecal content)	225 ± 21^c^	269 ± 14^b^	310 ± 29^a^
IgA (mg/ml serum)	4.525 ± 0.688^b^	5.145 ± 0.210^ab^	5.601 ± 0.551^a^
IgE (ng/ml serum)	507.7 ± 34.8	521.3 ± 30.2	539.9 ± 40.7
IgG (mg/ml serum)	8.198 ± 0.772^b^	8.627 ± 0.388^b^	8.853 ± 0.310^a^
IgM (mg/ml serum)	1.448 ± 0.169	1.502 ± 0.155	1.496 ± 0.109

Values are the mean ± standard division of six replicates. ^a–c^Means labelled with different superscripts within a row differ significantly (*P* < 0.05).

**Table 5:** Effect of OligoPKC on bacterial level in the cecum of rats (log⁡10 of cell/g cecal content).

Target bacterial group	Control	0.5% OligoPKC (L)	1% OligoPKC (H)
Total bacteria	9.17 ± 0.25^b^	9.80 ± 0.13^a^	9.87 ± 0.25^a^
*Lactobacillus*	8.20 ± 0.22^b^	8.62 ± 0.12^a^	8.83 ± 0.19^a^
*Bifidobacterium*	5.72 ± 0.24^b^	6.26 ± 0.21^a^	6.40 ± 0.38^a^
*Enterococcus *	6.38 ± 0.60^b^	7.03 ± 0.19^a^	6.90 ± 0.15^a^
*E. coli*	6.12 ± 0.43^a^	5.49 ± 0.58^b^	5.26 ± 0.44^b^
*Enterobacteriaceae*	6.49 ± 0.39^a^	6.07 ± 0.24^b^	5.97 ± 0.27^b^
Total *Salmonella*	4.23 ± 0.07^a^	3.84 ± 0.10^b^	3.76 ± 0.07^b^
Clostridiaceae	7.86 ± 0.23^a^	7.54 ± 0.16^ab^	7.24 ± 0.28^b^
*Campylobacter *spp.	3.17 ± 0.13	3.12 ± 0.07	3.16 ± 0.20

Values are the mean ± standard division of six replicates. ^a-b^Means labelled with different superscripts within a row differ significantly (*P* < 0.05).
